# Pathogenic Stress Induces Human Monocyte to Express an Extracellular Web of Tunneling Nanotubes

**DOI:** 10.3389/fimmu.2021.620734

**Published:** 2021-02-19

**Authors:** Michal Shahar, Auryan Szalat, Haim Rosen

**Affiliations:** ^1^The Department of Microbiology and Molecular Genetics, Institute for Medical Research-Israel-Canada, Hebrew University - Hadassah Medical Center, Jerusalem, Israel; ^2^Department of Internal Medicine, Hadassah-Hebrew University Medical Center, Jerusalem, Israel; ^3^The Kuvin Center for the Study of Infectious and Tropical Diseases, Institute for Medical Research-Israel-Canada, Hebrew University - Hadassah Medical School, Jerusalem, Israel

**Keywords:** monocyte, tunneling nanotubes, pathogen-associated molecular pattern, innate immunity, toll like receptor

## Abstract

Actin-based tunneling nanotubes are a means of intercellular communication between remote cells. In the last decade, this type of nanotube was described in a wide variety of cell types and it became widely accepted that communication through these nanotubes is related to response to environmental changes. Few reports, however, are available regarding the expression of similar nanotubes *in vivo* or in primary cells. Moreover, the functional significance of this intercellular communication for health and disease is largely unknown. In this context, and as a first step in unraveling these questions, we examined the formation of similar nanotubes in primary peripheral human monocytes. To that end, we combined the use of a live cell imaging system along with advanced methods of fluorescent and scanning electron microscopy. This experimental approach reveals for the first time that the bacterial lipopolysaccharide endotoxin induces a transient expression of an unexpected abundance of actin-based tunneling nanotubes associated with vesicles. In addition, it was found that a similar response can be achieved by treating human monocytes with various bacterial and yeast membrane components, as well as with a viral component analog. In all these cases, this response is mediated by distinct complexes of toll-like receptors. Therefore, we suggest that the observed phenomena are related to a broad type of monocyte pathogen response, and raise the possibility that the phenomena described above may be involved in many clinical situations related to inflammation as a new topic of study.

## Introduction

Cell-to-cell communication is fundamental to the existence and survival of multicellular organisms and occurs in different ways: indirect communication via intercellular messengers that travel for short or long distances, or direct communication via contact between adjacent cells. In a pioneering 2004 study, a new intercellular communication method between remote cells using actin-based tunneling nanotubes (TNTs) was described in rat PC12, normal rat kidney, and human embryonic kidney cells using three dimensional live-cell microscopy, scanning electron microscopy (SEM), and transmission electron microscopy (TEM) (1). Same year, TNT phenomenon was described in immune cells, including human peripheral blood NK cells, macrophages, and EBV-transformed B cells (2). Since then, TNTs have been described in additional immune cells, such as B cell line (3), T cells (4, 5), NK cells (6), human derived monocytic cell line THP1 and monocyte-derived dendritic cells (7) as well as murine macrophage cell lines (8, 9). TNTs were also observed in wide variety of malignant tissue-derived cell lines (10–16), in endothelial (17), epithelial lung (18) and mesenchymal stem cell populations (19, 20). Similarly, TNTs were found in primary cultures of mouse neuronal cells (21, 22) and human brain pericytes (23). TNTs have several specific characteristics and are highly heterogeneous in both structure and function: they are ultrafine intercellular structures spanning tens to hundreds of microns, a length of up to several cellular diameters and a width ranging from 50 to 1,500 nm (24, 25). They are membranous extensions from one cell to the other, supported by cytoskeletal fibers including F-actin and/or microtubules allowing unidirectional and/or bidirectional transmission of cellular components, a phenomenon known as “cargo transport,” for review, see (26–28). Recently, a comprehensive study led by Zurzolo (29) described the structural characterization of neuronal TNTs as a bundle of open-ended nanotubes bridged by threads of the cell adhesion molecule N-cadherin. Furthermore, high resolution analyses reveal that TNTs contain parallel actin bundles on which different cargoes appear to transfer (29). It is accepted that TNTs are stretched above the substratum between interconnected cells and rarely display a branched appearance. The involved mechanisms in TNT formation are complex, but distinct from those occurring in the formation of filopodia and cytonemes (24, 25, 30).

It seems that communication through TNTs is related to response to environmental changes, as it has been shown that TNTs are expressed transiently in a cell type-specific manner in response to a variety of stressors, including various products of pathogenic origin (30). Only a few reports are available regarding TNT expression in monocyte-derived differentiated cells and in the monocyte derived cell line THP1 and always in cell culture; in most cases, these are in the context of propagation of viruses as HIV (31–33), HSV (34), HTLV-1 (35), Porcine RSV (36) and transmission of prion protein as well (37).

However, despite ubiquitous observation of TNTs in cell cultures, only few studies described this phenomenon *in vivo* or *in situ*: Weil et al. (38) used glioblastomas cell lines implanted in chronic cranial windows in mice which developed TNTs participating in the propagation of the tumor cells and in the resistance to standard treatments; Parker et al. (39) studied TNTs formation in a model of brain metastases from human breast cancer cells injected in mice using lattice-light sheet microscopy and Alarcon- Martinez et al. (40) showed, TNTs connecting pericytes on separate blood vessels in living mouse retina which serve as a conduit for intercellular Ca^++^ waves under both physiological and pathological conditions.

Therefore, functional *in vivo* significance of these new intercellular communication phenomena in health and disease requires further evaluation. In this context, we examined TNT formation in peripheral human monocytes (PHMs). This immune cell type constitutes 4–10% of all peripheral leukocytes, and is characterized by expression of CD14 antigen, which is a co-receptor for toll-like receptor 4 (TLR4) which mediates lipopolysaccharide (LPS) signaling. The known role of monocytes and their derivatives is to defend against a variety of pathogens by their ability to recognize “danger signals,” among them pathogen-associated molecular pattern (PAMP) molecules, via pattern recognition receptors, presentation of antigens, secretion of chemokines, and proliferation in response to infection and injury (41, 42).

In the present study, we combine the use of a live cell imaging system, allowing us to continuously monitor TNTs formation in response to LPS in PHMs, with advanced fluorescent microscopy and SEM methods to further characterize the unique TNT ultrastructure. The results of this experimental approach demonstrate tremendous heterogeneity, complexity, and an unexpected abundance of membranous TNTs associated with vesicles. In addition, we find that the ability to induce TNT formation in PHMs is common to bacterial and yeast membrane components and a viral component analog as well, suggesting that the observed phenomena are related to a broad type of monocyte pathogen response mediated by distinct TLR complexes.

## Materials and Methods

### Reagents

LPS - Lipopolysaccharides from Escherichia coli O111:B4, Calcium Chloride solution 1 M in H_2_O and cytochalasin D from Sigma-Aldrich; EM Grade Paraformaldehyde (PFA) 16% and Gluteraldehyde 8% from Electron Microscopy Sciences; colchicine and colcemide 1 μg/mL in phosphate buffered saline from Biological Industries; **TLR agonists/antagonist** (in endotoxin-free water) from Invivogen: PAM3CSK4 – TLR2-TLR1 ligand, synthetic bacterial lipopeptide; FSL-1 – TLR2/TLR6 ligand, synthetic mycoplasmal lipoprotein; CL097 – TLR7/8 ligand, imidazoquinoline compound; LPS-RS Ultrapure – TLR4 antagonist, Ultrapure lipopolysaccharide from *Rhodobacter sphaeroides*; pHrodo™ Red Zymosan Bioparticles® Conjugate for phagocytosis from ThermoFisher Scientific.

### PHM Isolation

#### All Primary Material Was Used With Informed Consent in Accordance With the Declaration of Helsinki (16/LO/2055)

PHMs were isolated from whole blood of healthy consented volunteer donors using the EasySep™ Direct Human Monocyte Isolation Kit (Stemcell Technologies), according to the manufacturer's protocol, by immunomagnetic negative selection to achieve functional, untouched, highly purified CD14+ monocytes. The purity of the isolated monocytes was quality-controlled by the Cytoflex flow cytometer (Beckman Coulter) using an Alexa Fluor® 488 anti-human CD14 monoclonal antibody (clone HCD14) vs. Alexa Fluor® 488 Mouse IgG1 (clone MOPC-21), both from BioLegend. The purity of plastic-adhered monocytes was validated by immunofluorescence microscopy (IFM) using the same antibodies, showing CD14 presence in all cells.

### Cell and Tissue Culture

Freshly isolated cells were re-suspended in RPMI-1640 medium with L-glutamine and sodium bicarbonate (Sigma-Aldrich) adjusted to 1.2 mM final Ca^2+^ concentration and supplemented with 10% heat-inactivated fetal calf serum, 100 U/mL penicillin, and 100 μg/mL streptomycin (Gibco-Invitrogen), and cultured for up to 14 h in 5% CO_2_ at 37°C, as described (43).

### Microscopy Techniques

#### IncuCyte® S3 Live-Cell Analysis System

Twenty-five thousand PHMs per well were plated in Costar® 96-well flat bottom, tissue-culture (TC) treated, nonpyrogenic polystyrene sterile plates (Corning), and imaged within 15–45 minutes (min) of plating using the phase contrast channel in the IncuCyte® S3 (Essen BioScience, Inc.) platform, which was housed inside a cell incubator at 37°C with 5% CO_2_. Nine image sets from distinct regions per well were taken every hour using a 20x dry objective lens, and each condition was run in duplicate. IncuCyte software was set to recognize TNTs and calculate total nanotube length per area using the optimized NeuroTrack software module (Essen BioScience, Inc.) using the following analysis definition for phase neurites: “brightness” cell-body cluster segmentation mode with segmentation adjustment 1.4, minimal cell width 7 μm, “better” filtering for neurite parameters with neurite sensitivity of 0.75 and minimal neurite width to exclude objects above width of 1 μm. We could observe that peak TNTs density ranged from 3 to 6 h after exposure to LPS on the basis of live imaging by IncuCyte programmed to recognize TNTs structures, evaluating density of TNTs calculated by length of TNTs/area unit. Similar results were observed when density of TNTs were calculated by length of TNTs/cell bodies area (data not shown) to exclude the possibility that variations in TnTs were due to changes in number of cells.

#### Fluorescence Microscopy

Fluorescence microscopy was performed as previously described (44) with modifications. Freshly isolated PHMs were seeded in culture medium in Cellview glass bottom, TC-treated, 8-compartment dishes (Greiner Bio One International) at 50,000 cells per compartment, and fixed at the time of peak density of TNT expression (3–6 h).

TNT membrane components were directly labeled in living cells. Membrane-staining dyes were applied according to manufacturer's protocol. Briefly, 5 μg/mL wheat germ agglutinin (WGA) Alexa Fluor® 647 conjugate (ThermoFisher Scientific) was applied directly in culture medium at 4 h for 10 min at 37°C. After a brief wash with warm PBS, post-labeling fixation was performed using 4% PFA for 15 min at room temperature (RT). Alternatively, freshly isolated cells were labeled with 1 μM DiD (ThermoFisher Scientific) for 10 min at 37°C in serum-free media. Labeled cells were centrifuged for 5 min at 1,500 rpm, resuspended, and seeded as described above. Fluorescent signal was visualized using an Olympus IX71S8F inverted microscope equipped with a Retiga SRV fast 1394 (QImaging) cooled CCD camera and a 100x-1.4NA (Olympus) objective. Unlabeled TNTs were used to determine autofluorescence.

#### Spinning Disk Confocal Microscopy

TNT structural components and cargo were analyzed using indirect immunofluorescent (IF) labeling. Cells were fixed with Karnovsky fixative (2% PFA, 2.5% glutaraldehyde in 0.1 M cacodylate buffer, Ph = 7.4) for 30 min at RT. Samples were quenched with 50 mM NH_4_Cl for 15 min and permeabilized with 0.25% TWEEN® 20 in PBS for 5 min at RT. Blocking of non-specific binding was performed with 2% BSA in PBS for 20 min at RT. Specific antibodies were applied in 2% BSA/PBS overnight at 4°C at the following concentrations: 1:200 for anti-alpha smooth muscle actin antibody (α-SMA, ab5694, Abcam) and anti-α tubulin (clone B-5-1-2, Sigma-Aldrich); 5 μg/mL for anti-ATPIF1 antibody (5E2D7, ThermoFisher Scientific). Secondary antibodies (Cy™3-conjugated AffiniPure F(ab')_2_ Fragment Goat anti-Mouse IgG (Jackson ImmunoResearch) or Goat anti-Mouse IgG (H + L) Cross-Adsorbed Secondary Antibody, Alexa Fluor® 647 (ThermoFisher Scientific)) were diluted 1:200 in 2% BSA/PBS and added for 20 min at RT. For staining of nuclei and stabilization of fluorescent signals the samples were covered with Fluoroshield mounting medium containing DAPI (F6057, Sigma). Cells were visualized by Nikon Ti2E confocal fluorescent microscope with Yokogawa W1 Spinning Disk integrated with 50 μm pinhole, at 580 and 647 nm using a 60x-0.95NA CFA Plan-Apochromat Lambda objective. For 3D analysis, 25–38 Z-stack images of 0.5–0.7 μm width from the bottom to the top of the cells were acquired and processed by volume view extension of NIS Elements software package (Nikon). Unlabeled TNTs were used to determine autofluorescence.

### High Resolution SEM

At the peak time point of TNT expression (3–6 h), samples were fixed with Karnovsky's fixative for 4 h at RT then with 1:2 diluted Karnovsky's fixative overnight at 4°C, post-fixed in 1% OsO_4_ in 0.1 M cacodylate buffer for 2 h, dehydrated in a graded series of alcohols, dried in a critical point dryer (Quorum technology), and coated by Pd/Au (Quorum Technology) with coating thickness ~7 nm. The samples were imaged in a Sirion (FEI) Schottky field emission scanning electron microscope at an accelerating voltage of 5 kV using the secondary electron signal. Measurements of nanotube length and width were analyzed by XL Docu software.

Diameter of TnTs and vesicles was measured by normalizing the scale bar from the images to the number of pixels.

The research was approved by our institutional review board and all healthy volunteers who participated to the study signed informed consent forms.

## Results

### LPS-Stimulated PHMs Generate a Distinct Intercellular Network of TNTs

**Figure 1:**
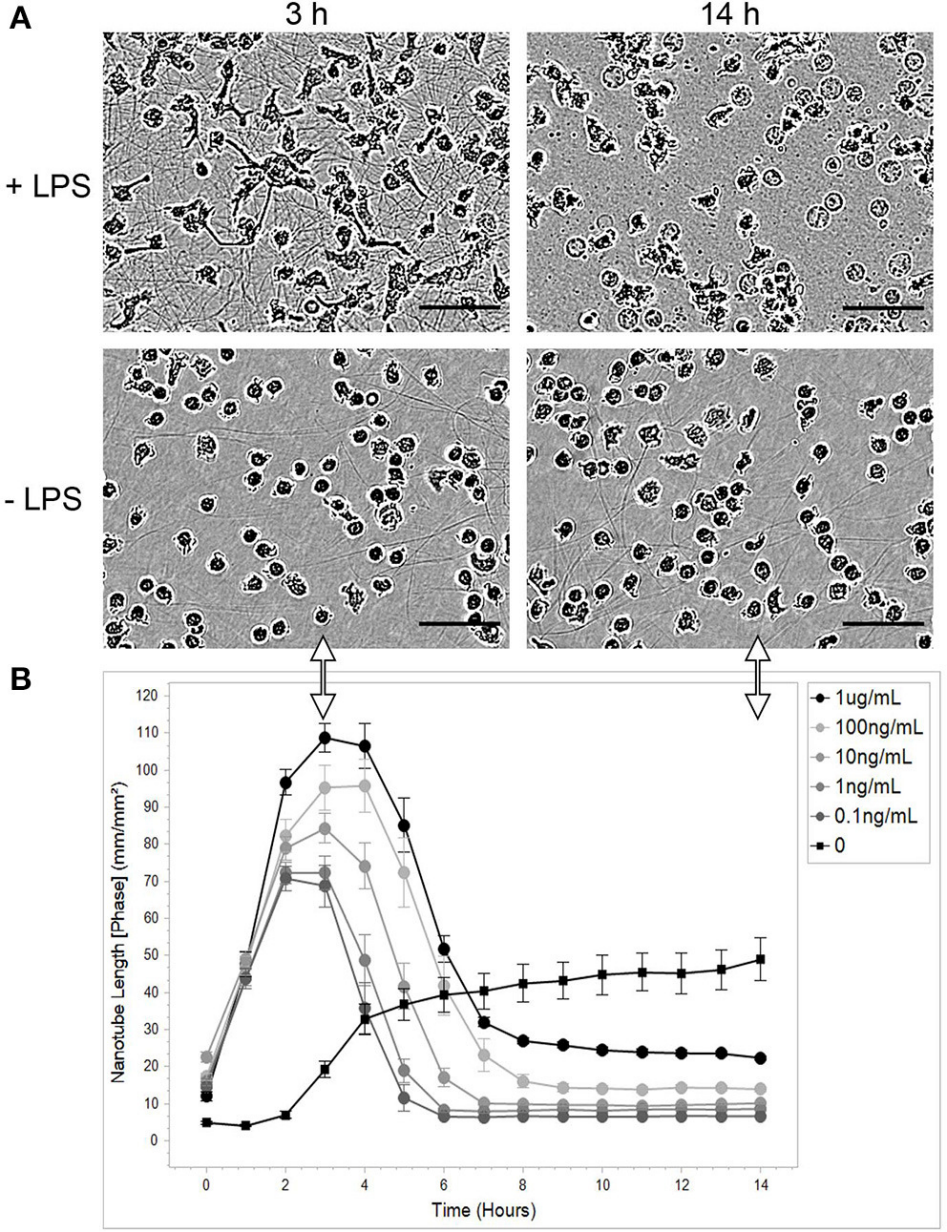
LPS causes PHM to transiently express TNTs. Monocytes were isolated, purified, and cultured as described in the Materials and Methods sections describing PHM isolation and cell culture. Cells were exposed to different concentrations of LPS and continuously monitored by IncuCyte® S3 Live-Cell Analysis System as described in Materials and Methods (Microscopy Techniques: IncuCyte® system). **(A)** Representative images of TNT abundance at peak time (3 h) and at the end of the experiment (14 h) from PHMs treated with 1 μg/mL LPS (+LPS) compared to untreated cells (-LPS). The images were obtained using a phase channel. The scale bar is 50 μm. **(B)** Quantification of mean nanotube length/area vs. time was obtained as described in Materials and Methods. The different LPS concentrations applied indicated in the top-right inset. Error bars are ± standard error of measurement (SEm), *n* = 9.

The aim of the present study was to investigate the possible expression of TNTs in PHMs. Since it is well known that a major obstacle in monitoring the formation of TNTs is their fragility, we chose to employ the IncuCyte live cell imaging system for tracking TNT-like structure formation. Images were registered every 1 h for 14 h. First, the ability of the stressor LPS, also known as endotoxin, to induce the expression of TNTs was examined (Figure 1). We observed that LPS induces the appearance of intercellular TNTs in a dose-dependent manner. The lowest LPS concentration that induced TNT appearance was 0.1 ng/mL. The quantitative index for TNT appearance was determined by the total length of these TNTs divided by an area unit. The kinetics of TNT appearance showed a bell-like shape with a peak (109 mm/mm^2^ at LPS 1 μg/mL) measured at 3–6 h after adding LPS to the cell culture medium (Figure 1B). TNT structures observed without LPS were similar to those found after LPS treatment, but differed significantly in their lower amount and slower kinetics of formation (19.2 mm/mm^2^, 5.7-fold less at 3 h than with LPS 1 μg/mL, Figure 1B).

Unexpected high density and amount of TNTs were observed (Figure 1B). These novel results can be attributed to the use of a real-time autofocus system that automatically captures and analyzes images of living cells while the cells remain undisturbed inside a standard tissue culture incubator. Another possibility is that the described phenomenon is monocyte-specific. Obviously, such surprising results required further testing in an independent optical system.

### The Intercellular Network Contains Heterogeneous TNTs

Initially, the identification of TNT-like structures in cell culture was based on the morphological criteria defined for PC12 cells (1). Subsequently, it has been found that a variety of TNTs exist, especially in immune cells (2, 45). Present criteria for TNT-like structures are: (a) they hover in the medium having no contact with the substratum and are capable of forming cell-to-cell connections; (b) they are long, thin membranous structures that mediate membrane continuity between connected cells; (c) they are 50–1,500 nm wide, spanning tens to hundreds of microns; (d) based on the cytoskeleton proteins identified in various TNTs, there are at least two types of TNTs, one characterized by the presence of F-actin and the other by the presence of both F- actin and tubulin; and (e) they allow both bi- and unidirectional transfer of information.

**Figure 2:**
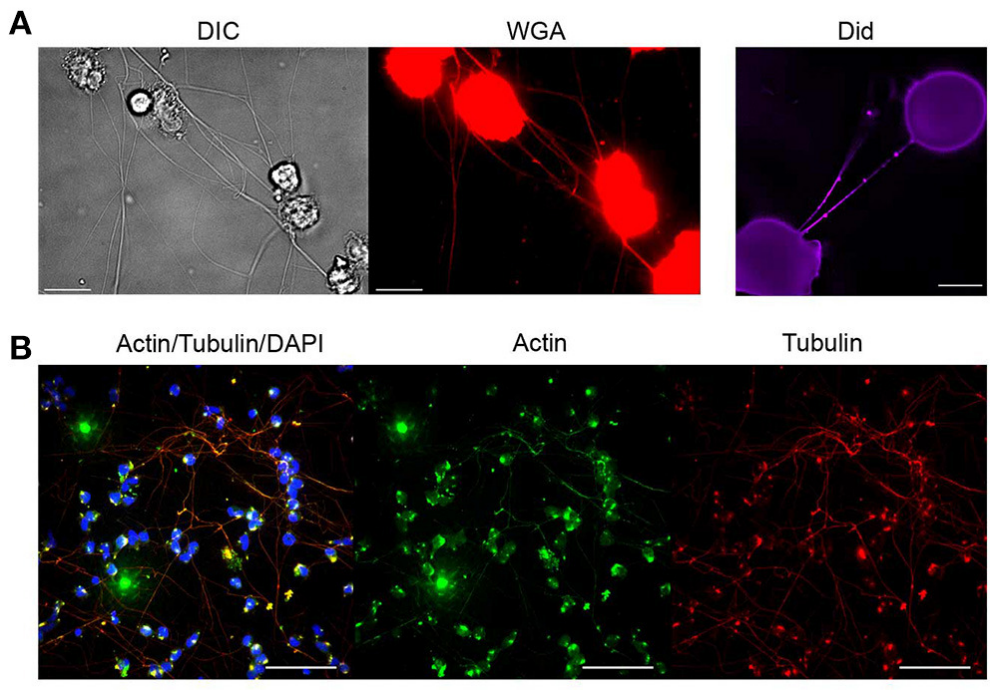
Molecular characterization of TNTs derived from monocytes. Characterization of TNTs was performed using fluorescence microscopy as described in Materials and Methods (Microscopy Techniques: Fluorescence microscopy). **(A)** Representative images show PHM cells stained with WGA or Did after 4 h of LPS treatment (1 μg/mL). Scale bar is 10 μm. **(B)** Confocal imaging of α-SMA (green), α-tubulin (red), and DNA (DAPI, blue) staining performed as described in Materials and Methods (Microscopy Techniques: Spinning disk confocal microscopy). Images were captured as Z-stacks (total 38 slices of 0.7 μm each) at 60x and are displayed as maximum intensity projection. All images are representative of at least two independent experiments performed in duplicate. Scale bar is 25 μm.

In view of these criteria and in order to elucidate the basic nature of PHM-derived TNTs, we used fluorescence and scanning electron microscopy. First, live labeling procedures with fluorescent wheat germ agglutinin (WGA) and DiD were applied to stain and delineate the plasma membrane of the newly observed TNTs. Results obtained after labeling with these reagents are depicted in Figure 2A, demonstrating above-surface cell-to-cell connections and membrane continuity between these cells. It is worth noting that membrane labeling reveals bead-like areas indicating possible cargo passing through the nanotube.

Subsequently, the expression of actin and tubulin, major cytoskeleton components and typical features of TNTs, was examined in the PHM-derived TNTs. Results of IF confocal microscopy with anti-tubulin and anti-actin specific antibodies are shown in Figure 2B, demonstrating that in most of the TNTs, there is co-expression of α-tubulin and α-smooth muscle actin. It is important to note that staining with phalloidin (high-affinity F-actin probe), widely used in TNTs studies, successfully stained cell bodies and filopodia-like short extensions, but failed to stain PHM TNTs (not shown).

**Figure 3:**
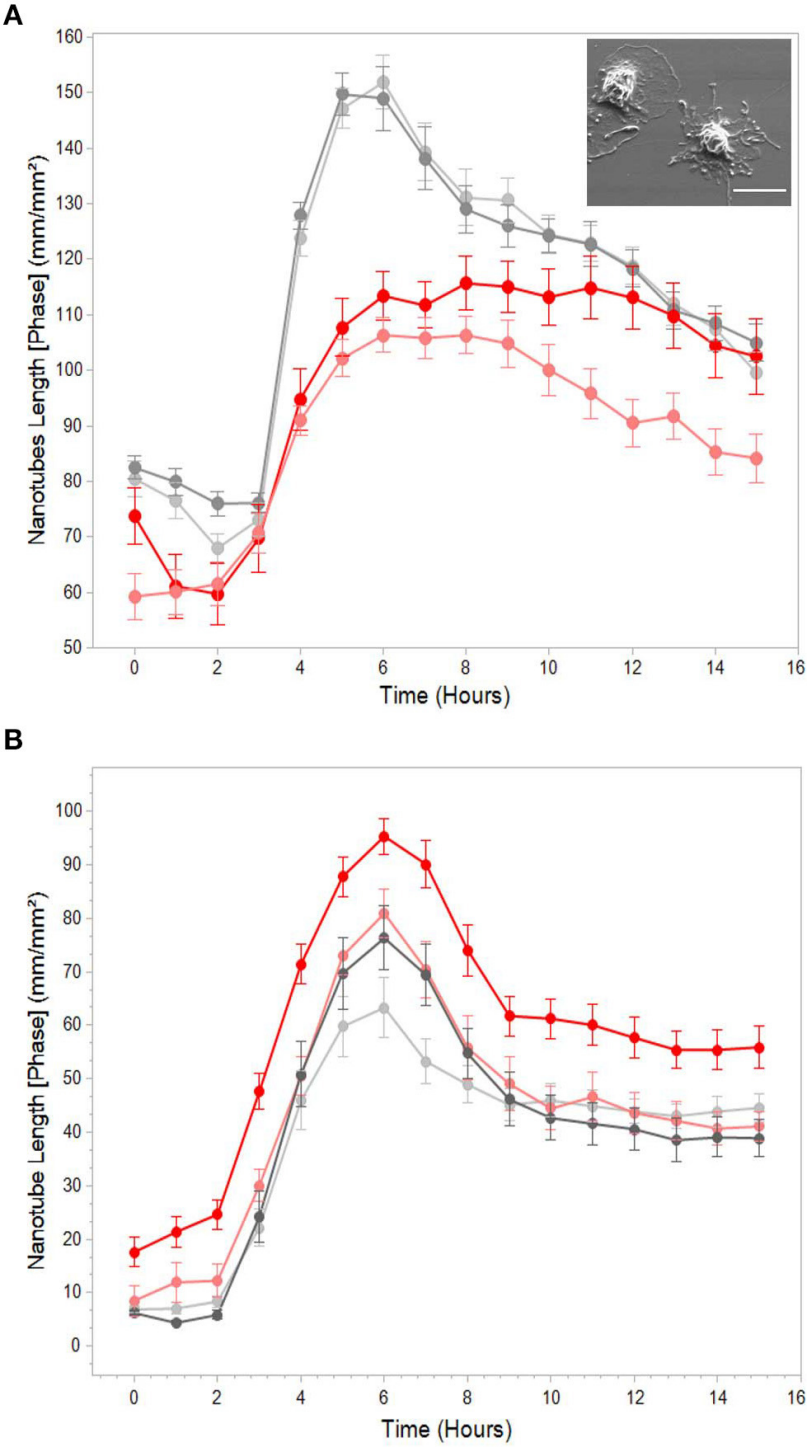
Effects of actin and tubulin polymerization inhibitors on LPS-induced TNTs. Representative graphs of total nanotube length vs. time with (red) or without (gray) pre-treatment with **(A)** actin polymerization inhibitor cytochalasin D (1 and 2 μM) and **(B)** tubulin polymerization inhibitors colchicine and colcemide (1 μg/mL both). TNT expression was live-monitored as in Figure 1, and treatment with inhibitors was performed as described in Materials and Methods (Microscopy Techniques: IncuCyte® system). The graphs highlight that cytochalasin D causes a decrease in TNT formation, whereas tubulin inhibitors (colchicine and colcemide) had no effects. **Inset:** typical morphology of PHMs treated with 2 μM cytochalasin D for 3 h. Scale bar is 10 μm. Error bars are ± SEm, *n* = 9, representative of 3 independent experiments.

**Figure 4:**
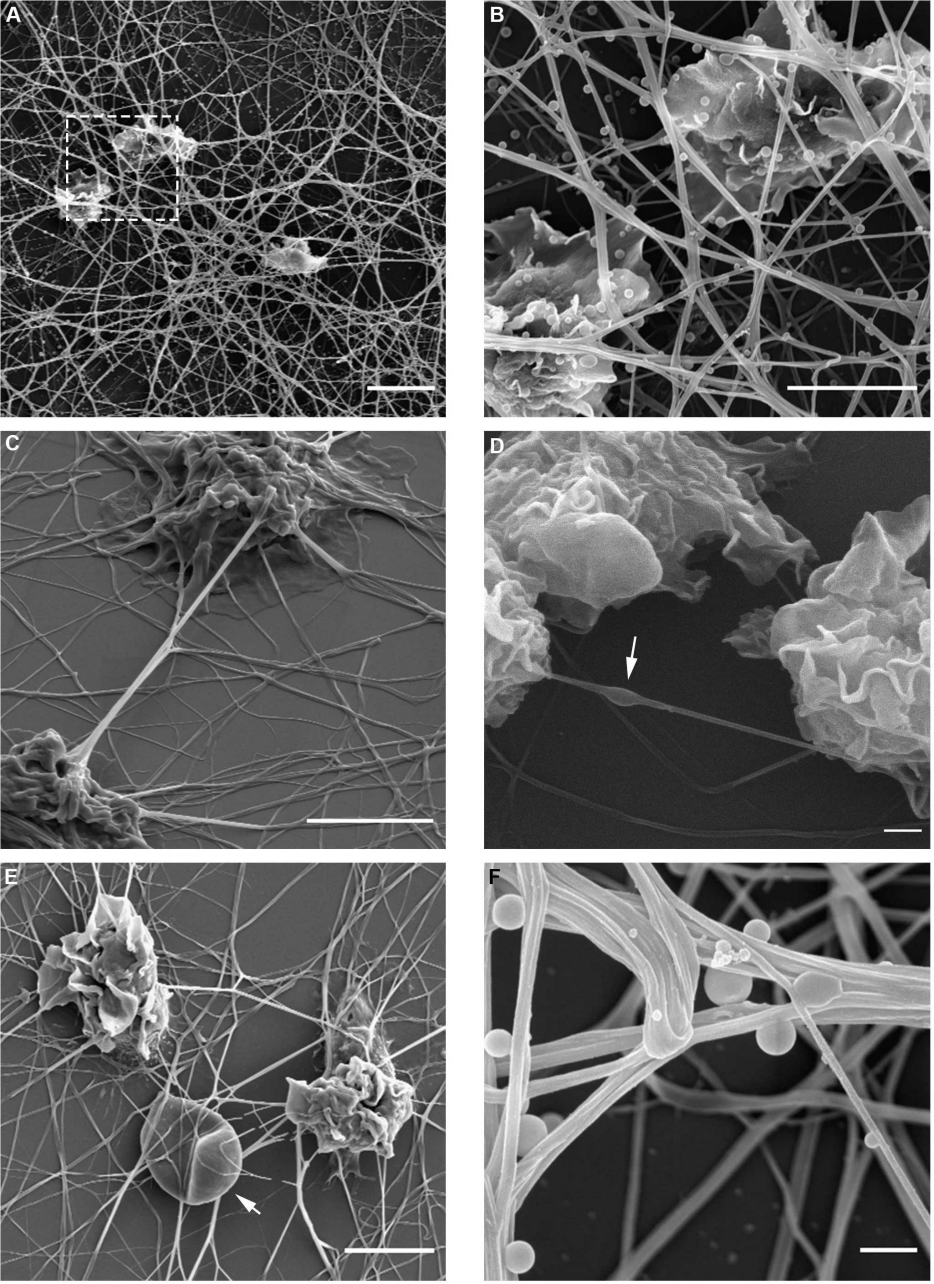
Spatial organization of LPS-induced TNTs in PHM culture. TNT heterogeneity and complexity was investigated using high resolution SEM in magnifications ranging 1,250–20,000. Magnified image of boxed area in **(A)** shown to the right **(B)**. Arrows indicate **(D)** bulges/gondola in TNT, **(E)** a trapped RBC, and **(F)** attached vesicles. Images were captured by scanning electron microscope as detailed in Materials and Methods (Microscopy Techniques: High resolution SEM). Scale bars are 10 μm **(A)**, 5 μm **(B–E)** and 0.5 μm **(F)**.

At this point, it warranted to test the effect of cytoskeleton-specific inhibitors on TNT expression. The effects of the actin polymerization inhibitor cytochalasin D on the LPS-induced expression of TNTs is shown in Figure 3A, whereas those of the tubulin polymerization inhibitors colchicine and colcemide are shown in Figure 3B. TNT expression was live-monitored using the IncuCyte platform as described above (see, Materials and Methods, Microscopy Techniques, IncuCyte® S3 live-cell analysis system), and the inhibitory reagents were added to the media together with the LPS (15 min after seeding to allow attachment). The results demonstrate that inhibition of tubulin polymerization into microtubules has no effect on the expression of PHM-derived TNTs (Figure 3B), whereas inhibition of actin polymerization into microfilaments inhibits some 50% of TNT expression (Figure 3A). The upper inset of Figure 3A demonstrates a typical PHM after 3 h of cytochalasin D (1 and 2 μM) treatment. These cells are characterized by a broad, flat shape and short TNT like-structures that do not form cell-to-cell connections and terminate in spherical-like structures, as predicted by a simulation of TNTs without actin cytoskeletons (46). To get better insight into the physical dimensions, heterogeneity, and three-dimensional organization of the LPS-induced TNTs, we turned to SEM. There are two main advantages in using this microscope: SEM micrographs have a large field depth, yielding a typical three-dimensional appearance that improves the understanding of the surface structure of a sample, and SEM enables a wide range of magnifications with a very high resolution, which is useful for nanometric structure sizing. Figure 4 demonstrates representative SEM micrographs of LPS-induced TNTs. The most prominent and novel phenomena in these images are the high concentration and complexity of the TNT arrangement in space (Figures 4A,B,E,F) as well as vesicles of varying sizes up to 450 nm found attached to the TNTs (Figures 4A,B,F). In addition, thin TNTs up to 50 nm wide and thick TNTs, up to 650 nm wide (Figures 4B,F) can be clearly discerned with the majority being 50–150 nm. In general, the very thin TNTs are concentrated adjacent to the cell substrate, whereas the thicker TNTs are preferentially located in the higher levels of the intercellular space away from the cell culture substrate. Furthermore, in several examples, a thick nanotube appears in a bundle-like structure (Figures 4C,F) that splits at a certain point into several thinner TNTs in such a way that their location in space cannot be always described as a straight line and they curve along different planes. It is worth mentioning two special features: first, the presence of a special shape in the nanotube (Figure 4D, arrow), which was previously suggested to be the result of cargo passage and was named a gondola (46); second, a red blood cell (RBC) trapped within the TNT web (Figure 4E, arrow), suggesting interaction with blood components involved in general hematologic homeostasis.

**Figure 5:**
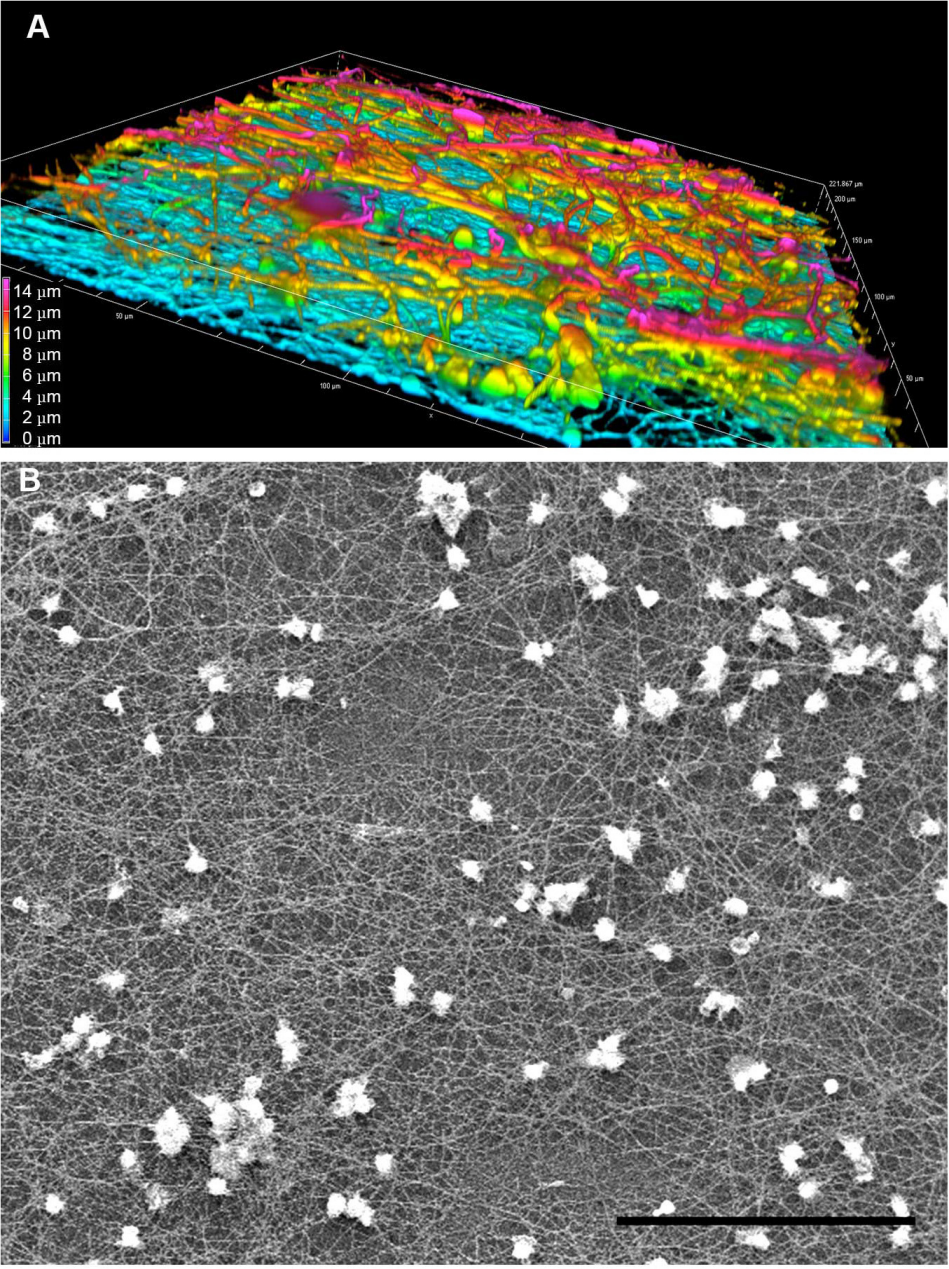
Complexity and heterogeneity of LPS-induced TNTs in PHM culture. Representative images obtained by **(A)** 3D confocal spinning disk IF with anti-α-tubulin antibody demonstrate multilayered nature of PHM-derived TNTs and **(B)** SEM at low magnification (500x) demonstrates large field depth of the TNT web. (**A**) shows a volume view with maximum intensity projection color-coded by depth, as produced by the NIS Elements software package. Imaging microscopy performed as described in Materials and Methods (Microscopy Techniques: spinning disk confocal microscopy and high resolution SEM, respectively). Scale bar is 15 μm **(A)** and 100 μm **(B)**.

To corroborate the findings presented in Figure 4, we probed the ultrastructure of LPS-induced TNTs using spinning disk confocal microscopy. Tubulin immunostaining enabled construction of a 3-dimensional, 15 micron-deep image (Figure 5A), indicating that many of these TNTs are not in contact with the underlying substratum. The volume view with maximum intensity projection color-coded by depth produced by the NIS Elements analysis software (Figure 5A) reveals a very complex and dense structure of straight and curved TNTs spanning from bottom to top, interconnecting to bundles and splitting again. This high level of complexity is well demonstrated by low-magnification SEM (Figure 5B).

### TNTs Contain Mitochondrial Cargo

**Figure 6:**
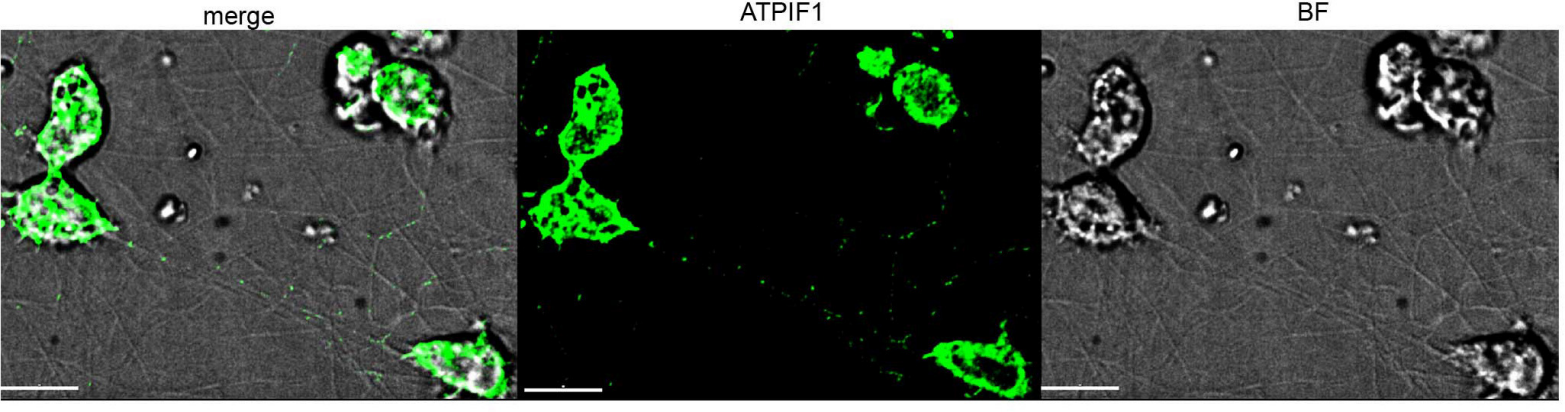
Mitochondrial transfer via TNTs. Confocal image of ATPIF1 staining performed as described in Materials and Methods (Microscope Techniques: Spinning disk confocal microscopy). Images were captured as Z-stacks (total 21 slices of 0.7 μm each) at 100x, and processed by AutoQuant Blind Deconvolution module. Fluorescent signals at Z-step number 10 are shown. The images are representative of two independent experiments. Scale bar is 10 μm.

A distinguishing feature of TNTs is their potential to transfer cellular components from cell to cell. To ensure that the structures observed here uphold this criterion, the presence of mitochondria was tested. To do so, cells were immuno-stained using an antibody for ATP synthase subunit IF1 (ATPIF1), a mitochondrial marker, as described in the Methods (Microscope Techniques: Spinning disk confocal microscopy). Confocal images clearly demonstrate IF signals along many TNTs (Figure 6), indicating the presence of mitochondria within the TNTs.

### Bacterial and Yeast Compounds Induce Intercellular TNT Networks

**Figure 7:**
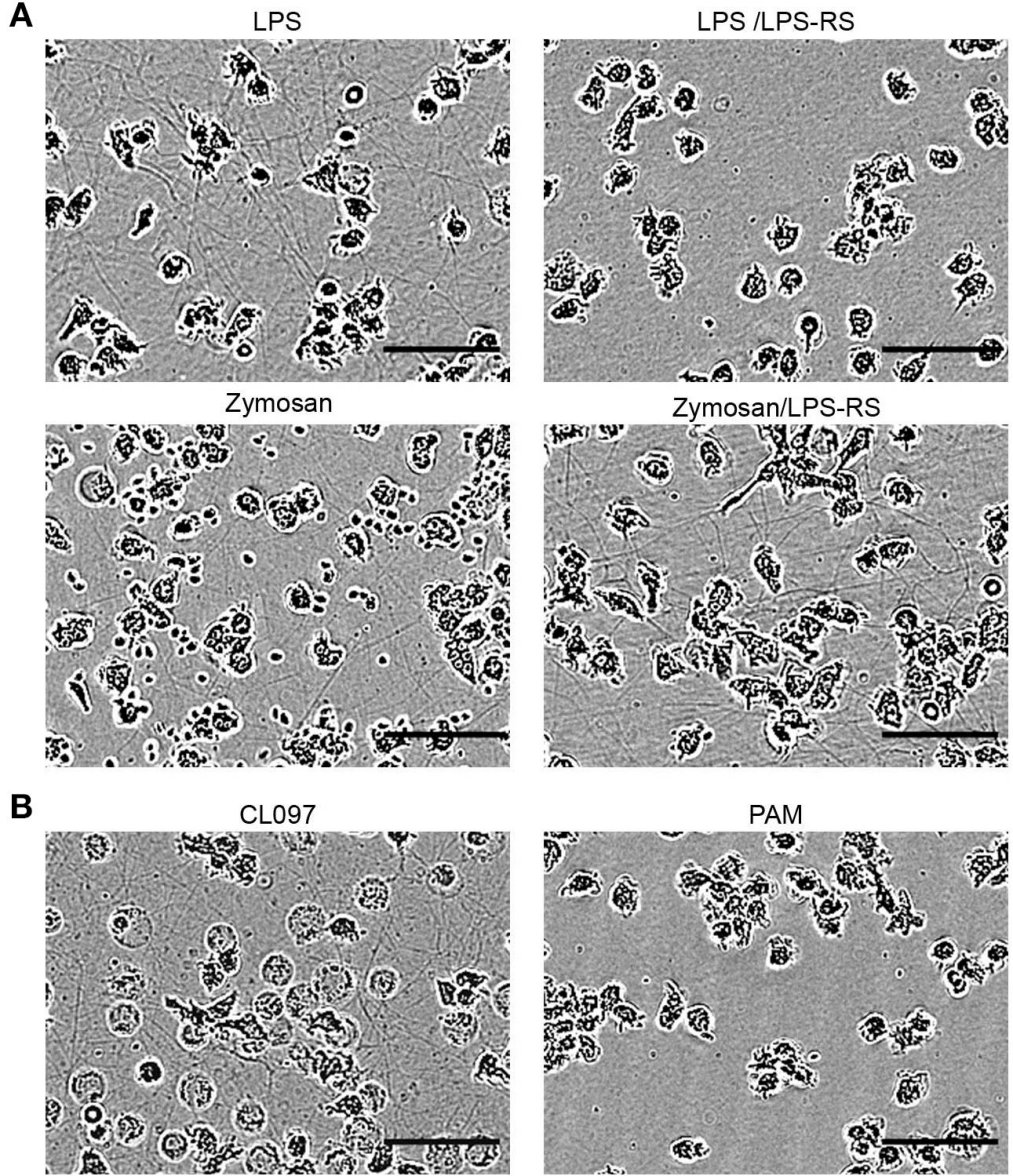
Involvement of defined TLRs in the formation of TNTs derived from monocytes. PMHs were exposed to different TLR agonists (LPS-RS, Zymosan, CL097, and PAM, as indicated), and continuously monitored as described in the Figure 1. **(A)** 10 μg/mL LPS-RS, a TLR4 antagonist, was added to PHMs 15 min prior to exposure to 10 ng/mL LPS. A similar procedure was followed with 10 μg/mL LPS-RS and 3 μg Zymosan, an insoluble preparation of cell wall from Saccharomyces cerevisiae, recognized by TLR2 and also by Dectin-1. **(B)** 1 μg/mL of the synthetic bacterial lipopeptide PAM3CSK4 (PAM), a TLR2/1 ligand, and 3 μg/mL of the imidazoquinoline compound CL097, a TLR7/8 ligand, were applied. Images show TNT abundance at peak density time point (4–8 h). All images are representative of at least two independent experiments performed in duplicate. Scale bar is 50 μm.

The innate recognition of microorganism-derived compounds or components is mediated by toll-like receptors (TLRs), which are genomically encoded pattern recognition receptors. The contribution of these receptors to TNT induction has not yet been studied. The central role of TLR4 in the LPS-induced PHM TNT induction mechanism was tested by applying a specific TLR4 antagonist (LPS-RS Ultrapure). Indeed, TNT formation was completely blocked (Figure 7A). The ability of additional stressors to induce the expression of TNTs was examined by applying agonists to TLRs known to be expressed in human monocytes (Figure 7). We observed that CL097, a viral component analog known to be a TLR8 agonist, is a strong inducer of TNTs (Figure 7B), but the agonists for TLR2/6 and TLR 2/1 (synthetic bacterial lipopeptide PAM3CSK4 (PAM), Figure 7B and synthetic mycoplasmal lipoprotein FSL-1 (not shown)) are not. CL097 induced TNTs in a dose-dependent manner (not shown), at a concentration range specific to TLR8 with similar kinetics and resulting TNT appearance as that resulting from LPS.

Surprisingly, another TLR2 agonist, Zymosan, typically applied for phagocytosis study, induced a similar TNT web, as revealed by IncuCyte imaging (Figure 7A). These Zymosan-induced TNTs were not affected by TLR4 blocking (Figure 7A), therefore excluding the possibility of LPS contamination as a TNT inducer in this case. Since other TLR2 agonists tested failed to induce expression of TNTs, a possible mechanism involved here is Dectin 1 mediated pathway (47).

## Discussion

Monocytes and neutrophils are central players in the innate immune response, which is the first line of host defense found in all multicellular organisms. As of today, a large body of knowledge has been accumulated demonstrating how these cells contribute to inflammation and host defense by pattern recognition receptor-mediated recruitment and cytokine release, phagocytosis, oxidative burst, and antigen presentation (42); novel studies show that macrophages and neutrophils can also release extracellular nucleic acids capable of entrapping exogenous pathogens, a phenomenon currently defined as METosis and NETosis, respectively (48, 49). Beyond the classic known mechanisms of action of monocytes-macrophages and the latter cited entrapment phenomenon, our study adds another new type of capacity for peripheric human monocytes and a new type of response: intercellular network of TNTs formed by stimulated circulating monocytes mediated by pattern recognition receptors. Intercellular network of TNTs formed by stimulated circulating monocytes mediated by pattern recognition receptors. This new characteristic needs to be further explored to understand its exact role in the whole inflammatory response of the mononuclear phagocyte system. TNTs phenomenon maybe participate in different functions of monocytes as tissue- repair and inflammation response. It also seems to offer the possibility of direct communication, maybe leading to coordinated activity, between involved monocytes.

A combination of microscopy techniques (live imaging, electron microscopy and immunofluorescence) allowed us to characterize the kinetics, molecular composition, and ultra-structure of TNTs in PHMs. We show that these TNTs correspond closely with previous descriptions, with some particularities: branched TNTs forming nets of TNTs between cells, cell bodies covered with a net-like structure, unique cytoskeleton composition, thick bundles composed of several TNTs, and two interconnected populations of TNTs formed preferentially at higher cell density: thin, substratum-adjacent TNTs, and TNTs hovering over the substratum of a wide diameter range (<1 μm). Findings obtained with selective cytoskeleton inhibitors indicated that only actin cytoskeleton integrity partially affected TNT formation, emphasized the heterogeneity of the observed nanotubes. The three-dimensional analysis by SEM and IFM further confirmed the complex and heterogeneous nature of these TNT structures at different depths (cell heights). Gondola-like structures and enclosed mitochondria were observed along these TNTs, suggestive of TNT function in intercellular communication. In addition, TNT-attached vesicles were also observed. Finally, a mechanism involving TLR4 and TLR8 was identified. Further molecular studies are needed to decipher the involved mechanisms evaluating the participation of concomitant pathways, such as the inflammasome via NLRP3 or the role of Dectin 1.

In other studies, TNTs were described in various cell lines including *in vitro* differentiated immune cells, among them macrophages prepared either from peripheral blood monocytes or bone-marrow derived mononuclear progenitor cells, by a 5–12 day cultivation procedure with or without differentiation-stimulating cytokines (2, 50, 51). The most studied *in vitro* role of TNTs in macrophages is the transfer of pathogens, suggesting a mechanism allowing pathogens spread without detection (33). Numerous studies showed mitochondrial transfer via TNTs *in vitro* (19, 20, 28, 51). However, until now, very few studies examined TNT formation in primary immune system cells, or in immune cells *in vivo*. To the best of our knowledge, there are only few published studies reporting *in vivo* formation of TNTs in mononuclear cells: in mouse cornea subjected to trauma and LPS, demonstrating antigen transmission between widely separated cornea dendritic cells via TNTs (52); in cystinotic mouse kidney transplanted with hematopoietic stem cells differentiated to macrophages, demonstrating lysosomal transfer between macrophages and diseased renal cells via TNTs crossing the tubular basement membrane (50). The difficulty to perform *in vivo* studies to observe TNTs is related to the fragility of these structures which necessitate strict environmental conditions and advanced technologies to be successful (25). The findings of our study provide the first evidence for the formation of membrane TNTs in freshly isolated, circulating human monocytes upon attachment to solid substrate and exposure to PAMP molecules. The density of TNTs is PHM/PAMP concentration- and time-dependent. At a high concentration of monocytes or stressors, we observed a web of TNTs so dense that it could possibly clog small blood vessels. The putative beneficial functions of the TNTs observed in PHMs, as well as of massive expression of TNTs associated with severe virulence require further investigation. A combination of PAMP molecules, recruited monocytes and their adhesion to vascular endothelium characterizes various pathologies, including injury, inflammation, atherosclerosis, septic shock, and virus-induced coagulopathy (53–55). Taken together, our study led us to raise the following questions: do PHM-specific TNTs have a role in some of these pathologies and could they serve as therapeutic target? Our study does not answer these questions, as we did not assess TNT formation in monocytes from patients with active diseases; however, it is tempting to assume that TNTs formation is a wide biologic phenomenon which may be involved in pathologic processes (infectious, oncologic, neurologic, and immunologic), and which should be further explored using adapted protocols.

## Data Availability Statement

The original contributions presented in the study are included in the article/supplementary material, further inquiries can be directed to the corresponding author/s.

## Ethics Statement

The studies involving human participants were reviewed and approved by Hadassah Medical Center Internal Review Board. The patients/participants provided their written informed consent to participate in this study.

## Author Contributions

MS performed all experiments with contributions from AS. AS and HR designed the project. MS, AS, and HR interpreted the data and wrote the manuscript. HR supervised all aspects of the work. All authors contributed to the article and approved the submitted version.

## Conflict of Interest

The authors declare that the research was conducted in the absence of any commercial or financial relationships that could be construed as a potential conflict of interest.

## References

[1] Rustom A Saffrich R Markovic I Walther P Gerdes HH. Nanotubular highways for intercellular organelle transport. Science. (2004) 303:1007–10. 10.1126/science.1093133 14963329

[2] Onfelt B Nedvetzki S Yanagi K Davis DM. Cutting edge: membrane nanotubes connect immune cells. J Immunol. (2004) 173:1511–3. 10.4049/jimmunol.173.3.1511 15265877

[3] Rainy N Chetrit D Rouger V Vernitsky H Rechavi O Marguet D. H-Ras transfers from B to T cells via tunneling nanotubes. Cell Death Dis. (2013) 4:e726. 10.1038/cddis.2013.245 23868059 PMC3730418

[4] Luchetti F Canonico B Arcangeletti M Guescini M Cesarini E Stocchi V. Fas signalling promotes intercellular communication in T cells. PLoS ONE. (2012) 7:e35766. 10.1371/journal.pone.0035766 22558220 PMC3338457

[5] Matula Z Nemeth A Lorincz P Szepesi A Brozik A Buzas EI. The role of extracellular vesicle and tunneling nanotube-mediated intercellular cross-talk between mesenchymal stem cells and human peripheral T cells. Stem Cells Dev. (2016) 25:1818–32. 10.1089/scd.2016.0086 27596268

[6] Chauveau A Aucher A Eissmann P Vivier E Davis DM. Membrane nanotubes facilitate long-distance interactions between natural killer cells and target cells. Proc Natl Acad Sci USA. (2010) 107:5545–50. 10.1073/pnas.0910074107 20212116 PMC2851811

[7] Watkins SC Salter RD. Functional connectivity between immune cells mediated by tunneling nanotubules. Immunity. (2005) 23:309–18. 10.1016/j.immuni.2005.08.009 16169503

[8] Bittins M Wang X. TNT-induced phagocytosis: tunneling nanotubes mediate the transfer of pro-phagocytic signals from apoptotic to viable cells. J Cell Physiol. (2017) 232:2271–9. 10.1002/jcp.25584 27591547 PMC5485076

[9] Hanna SJ McCoy-Simandle K Miskolci V Guo P Cammer M Hodgson L. The role of Rho-GTPases and actin polymerization during macrophage tunneling nanotube biogenesis. Sci Rep. (2017) 7:8547. 10.1038/s41598-017-08950-7 28819224 PMC5561213

[10] Lou E Fujisawa S Morozov A Barlas A Romin Y Dogan Y. Tunneling nanotubes provide a unique conduit for intercellular transfer of cellular contents in human malignant pleural mesothelioma. PLoS ONE. (2012) 7:e33093. 10.1371/journal.pone.0033093 22427958 PMC3302868

[11] Ady JW Desir S Thayanithy V Vogel RI Moreira AL Downey RJ. Intercellular communication in malignant pleural mesothelioma: properties of tunneling nanotubes. Front Physiol. (2014) 5:400. 10.3389/fphys.2014.00400 25400582 PMC4215694

[12] Antanaviciute I Rysevaite K Liutkevicius V Marandykina A Rimkute L Sveikatiene R. Long-distance communication between laryngeal carcinoma cells. PLoS ONE. (2014) 9:e99196. 10.1371/journal.pone.0099196 24945745 PMC4063716

[13] D'Aloia A Berruti G Costa B Schiller C Ambrosini R Pastori V. RalGPS2 is involved in tunneling nanotubes formation in 5637 bladder cancer cells. Exp Cell Res. (2018) 362:349–61. 10.1016/j.yexcr.2017.11.036 29208460

[14] Desir S Dickson EL Vogel RI Thayanithy V Wong P Teoh D. Tunneling nanotube formation is stimulated by hypoxia in ovarian cancer cells. Oncotarget. (2016) 7:43150–61. 10.18632/oncotarget.9504 27223082 PMC5190014

[15] Saenz-de-Santa-Maria I Bernardo-Castineira C Enciso E Garcia-Moreno I Chiara JL Suarez C. Control of long-distance cell-to-cell communication and autophagosome transfer in squamous cell carcinoma via tunneling nanotubes. Oncotarget. (2017) 8:20939–60. 10.18632/oncotarget.15467 28423494 PMC5400557

[16] Valdebenito S Audia A Bhat KPL Okafo G Eugenin EA. Tunneling nanotubes mediate adaptation of glioblastoma cells to temozolomide and ionizing radiation treatment. iScience. (2020) 23:101450. 10.1016/j.isci.2020.101450 32882515 PMC7476317

[17] Astanina K Koch M Jungst C Zumbusch A Kiemer AK. Lipid droplets as a novel cargo of tunnelling nanotubes in endothelial cells. Sci Rep. (2015) 5:11453. 10.1038/srep11453 26095213 PMC4476149

[18] Kumar A Kim JH Ranjan P Metcalfe MG Cao W Mishina M. Influenza virus exploits tunneling nanotubes for cell-to-cell spread. Sci Rep. (2017) 7:40360. 10.1038/srep40360 28059146 PMC5216422

[19] Vallabhaneni KC Haller H Dumler I. Vascular smooth muscle cells initiate proliferation of mesenchymal stem cells by mitochondrial transfer via tunneling nanotubes. Stem Cells Dev. (2012) 21:3104–13. 10.1089/scd.2011.0691 22676452 PMC3495124

[20] Liu K Ji K Guo L Wu W Lu H Shan P. Mesenchymal stem cells rescue injured endothelial cells in an *in vitro* ischemia-reperfusion model via tunneling nanotube like structure-mediated mitochondrial transfer. Microvasc Res. (2014) 92:10–8. 10.1016/j.mvr.2014.01.008 24486322

[21] Sharma M Subramaniam S. Rhes travels from cell to cell and transports Huntington disease protein via TNT-like protrusion. J Cell Biol. (2019) 218:1972–93. 10.1083/jcb.201807068 31076452 PMC6548131

[22] Costanzo M Abounit S Marzo L Danckaert A Chamoun Z Roux P. Transfer of polyglutamine aggregates in neuronal cells occurs in tunneling nanotubes. J Cell Sci. (2013) 126:3678–85. 10.1242/jcs.126086 23781027

[23] Dieriks BV Park TI Fourie C Faull RL Dragunow M Curtis MA. alpha-synuclein transfer through tunneling nanotubes occurs in SH-SY5Y cells and primary brain pericytes from Parkinson's disease patients. Sci Rep. (2017) 7:42984. 10.1038/srep42984 28230073 PMC5322400

[24] Davis DM Sowinski S. Membrane nanotubes: dynamic long-distance connections between animal cells. Nat Rev Mol Cell Biol. (2008) 9:431–6. 10.1038/nrm2399 18431401

[25] Mittal R Karhu E Wang JS Delgado S Zukerman R Mittal J. Cell communication by tunneling nanotubes: implications in disease and therapeutic applications. J Cell Physiol. (2019) 234:1130–46. 10.1002/jcp.27072 30206931

[26] Abounit S Zurzolo C. Wiring through tunneling nanotubes–from electrical signals to organelle transfer. J Cell Sci. (2012) 125:1089–98. 10.1242/jcs.083279 22399801

[27] Marzo L Gousset K Zurzolo C. Multifaceted roles of tunneling nanotubes in intercellular communication. Front Physiol. (2012) 3:72. 10.3389/fphys.2012.00072 22514537 PMC3322526

[28] Vignais ML Caicedo A Brondello JM Jorgensen C. Cell connections by tunneling nanotubes: effects of mitochondrial trafficking on target cell metabolism, homeostasis, and response to therapy. Stem Cells Int. (2017) 2017:6917941. 10.1155/2017/6917941 28659978 PMC5474251

[29] Sartori-Rupp A Cordero Cervantes D Pepe A Gousset K Delage E Corroyer-Dulmont S. Correlative cryo-electron microscopy reveals the structure of TNTs in neuronal cells. Nat Commun. (2019) 10:342. 10.1038/s41467-018-08178-7 30664666 PMC6341166

[30] Korenkova O Pepe A Zurzolo C. Fine intercellular connections in development: TNTs, cytonemes, or intercellular bridges? Cell Stress. (2020) 4:30–43. 10.15698/cst2020.02.212 32043076 PMC6997949

[31] Sowinski S Jolly C Berninghausen O Purbhoo MA Chauveau A Kohler K. Membrane nanotubes physically connect T cells over long distances presenting a novel route for HIV-1 transmission. Nat Cell Biol. (2008) 10:211–9. 10.1038/ncb1682 18193035

[32] Eugenin EA Gaskill PJ Berman JW. Tunneling nanotubes (TNT) are induced by HIV-infection of macrophages: a potential mechanism for intercellular HIV trafficking. Cell Immunol. (2009) 254:142–8. 10.1016/j.cellimm.2008.08.005 18835599 PMC2701345

[33] Hashimoto M Bhuyan F Hiyoshi M Noyori O Nasser H Miyazaki M. Potential role of the formation of tunneling nanotubes in HIV-1 spread in macrophages. J Immunol. (2016) 196:1832–41. 10.4049/jimmunol.1500845 26773158

[34] Panasiuk M Rychlowski M Derewonko N Bienkowska-Szewczyk K. Tunneling nanotubes as a novel route of cell-to-cell spread of herpesviruses. J Virol. (2018) 92:e00090–18. 10.1128/JVI.00090-18 29491165 PMC5923070

[35] Omsland M Pise-Masison C Fujikawa D Galli V Fenizia C Parks RW. Inhibition of tunneling nanotube (TNT) formation and human T-cell leukemia virus type 1 (HTLV-1) transmission by cytarabine. Sci Rep. (2018) 8:11118. 10.1038/s41598-018-29391-w 30042514 PMC6057998

[36] Guo R Katz BB Tomich JM Gallagher T Fang Y. Porcine reproductive and respiratory syndrome virus utilizes nanotubes for intercellular spread. J Virol. (2016) 90:5163–75. 10.1128/JVI.00036-16 26984724 PMC4859731

[37] Gousset K Schiff E Langevin C Marijanovic Z Caputo A Browman DT. Prions hijack tunnelling nanotubes for intercellular spread. Nat Cell Biol. (2009) 11:328–36. 10.1038/ncb1841 19198598

[38] Weil S Osswald M Solecki G Grosch J Jung E Lemke D. Tumor microtubes convey resistance to surgical lesions and chemotherapy in gliomas. Neuro Oncol. (2017) 19:1316–26. 10.1093/neuonc/nox070 28419303 PMC5596180

[39] Parker I Evans KT Ellefsen K Lawson DA Smith IF. Lattice light sheet imaging of membrane nanotubes between human breast cancer cells in culture and in brain metastases. Sci Rep. (2017) 7:11029. 10.1038/s41598-017-11223-y 28887508 PMC5591308

[40] Alarcon-Martinez L Villafranca-Baughman D Quintero H Kacerovsky JB Dotigny F Murai KK. Interpericyte tunnelling nanotubes regulate neurovascular coupling. Nature. (2020) 585:91–5. 10.1038/s41586-020-2589-x 32788726

[41] Auffray C Sieweke MH Geissmann F. Blood monocytes: development, heterogeneity, and relationship with dendritic cells. Annu Rev Immunol. (2009) 27:669–92. 10.1146/annurev.immunol.021908.132557 19132917

[42] Guilliams M Mildner A Yona S. Developmental and functional heterogeneity of monocytes. Immunity. (2018) 49:595–613. 10.1016/j.immuni.2018.10.005 30332628

[43] Rossol M Pierer M Raulien N Quandt D Meusch U Rothe K. Extracellular Ca2+ is a danger signal activating the NLRP3 inflammasome through G protein-coupled calcium sensing receptors. Nat Commun. (2012) 3:1329. 10.1038/ncomms2339 23271661 PMC3535422

[44] Abounit S Delage E Zurzolo C. Identification and characterization of tunneling nanotubes for intercellular trafficking. Curr Protoc Cell Biol. (2015) 67:12.0.1–12.10.21. 10.1002/0471143030.cb1210s67 26061240

[45] Dupont M Souriant S Lugo-Villarino G Maridonneau-Parini I Verollet C. Tunneling nanotubes: intimate communication between myeloid cells. Front Immunol. (2018) 9:43. 10.3389/fimmu.2018.00043 29422895 PMC5788888

[46] Drab M Stopar D Kralj-Iglic V Iglic A. Inception mechanisms of tunneling nanotubes. Cells. (2019) 8:626. 10.3390/cells8060626 PMC6627088 31234435

[47] Gantner BN Simmons RM Canavera SJ Akira S Underhill DM. Collaborative induction of inflammatory responses by dectin-1 and Toll-like receptor 2. J Exp Med. (2003) 197:1107–17. 10.1084/jem.20021787 12719479 PMC2193968

[48] Kazzaz NM Sule G Knight JS. Intercellular interactions as regulators of NETosis. Front Immunol. (2016) 7:453. 10.3389/fimmu.2016.00453 27895638 PMC5107827

[49] Doster RS Rogers LM Gaddy JA Aronoff DM. Macrophage extracellular traps: a scoping review. J Innate Immun. (2018) 10:3–13. 10.1159/000480373 28988241 PMC6757166

[50] Naphade S Sharma J Gaide Chevronnay HP Shook MA Yeagy BA Rocca CJ. Brief reports: lysosomal cross-correction by hematopoietic stem cell-derived macrophages via tunneling nanotubes. Stem Cells. (2015) 33:301–9. 10.1002/stem.1835 25186209 PMC4270893

[51] Jackson MV Morrison TJ Doherty DF McAuley DF Matthay MA Kissenpfennig A. Mitochondrial transfer via tunneling nanotubes is an important mechanism by which mesenchymal stem cells enhance macrophage phagocytosis in the *in vitro* and *in vivo* models of ARDS. Stem Cells. (2016) 34:2210–23. 10.1002/stem.2372 27059413 PMC4982045

[52] Chinnery HR Pearlman E McMenamin PG. Cutting edge: membrane nanotubes *in vivo*: a feature of MHC class II+ cells in the mouse cornea. J Immunol. (2008) 180:5779–83. 10.4049/jimmunol.180.9.5779 18424694 PMC3392179

[53] Ikeda U Takahashi M Shimada K. Monocyte-endothelial cell interaction in atherogenesis and thrombosis. Clin Cardiol. (1998) 21:11–4. 10.1002/clc.4960210103 9474460 PMC6655497

[54] Ingersoll MA Platt AM Potteaux S Randolph GJ. Monocyte trafficking in acute and chronic inflammation. Trends Immunol. (2011) 32:470–7. 10.1016/j.it.2011.05.001 21664185 PMC3179572

[55] Goeijenbier M van Wissen M van de Weg C Jong E Gerdes VE Meijers JC. Review: Viral infections and mechanisms of thrombosis and bleeding. J Med Virol. (2012) 84:1680–96. 10.1002/jmv.23354 22930518 PMC7166625

